# Gastric mucosal change after the eradication of *Helicobacter pylori* in patients at high risk for gastric cancers

**DOI:** 10.3389/fmed.2025.1679012

**Published:** 2025-11-26

**Authors:** Na Rae Lim, Woo Chul Chung

**Affiliations:** Department of Internal Medicine, The Catholic University of Korea, St. Vincent’s Hospital, Suwon, Republic of Korea

**Keywords:** gastric cancer, *Helicobacter pylori*, eradication therapy, endoscopic submucosal dissection, metachronous gastric cancer

## Abstract

**Background:**

Eradication of *Helicobacter pylori* alone will not eliminate the risk of gastric cancer. There was no conclusive evidence that eradication therapy reduced the incidence of gastric cancer in patients with atrophic gastritis or intestinal metaplasia.

**Materials and methods:**

This study was conducted from March 2010 to September 2022, and enrolled 1,789 consecutive patients who underwent endoscopic submucosal dissection for gastric mucosal dysplasia. Patients who underwent two or more procedures for simultaneous or metachronous gastric epithelial dysplasia as high-risk groups for gastric cancer, and were confirmed to have *H. pylori* infection at the time of the procedure were included. Patients who received eradication treatment and underwent endoscopic follow-up for at least 2 years were included. The control group included patients who underwent endoscopic treatment for simple low-grade dysplasia. We investigated whether endoscopy was performed between 1 and 2 years after successful eradication of *H. pylori.* The endoscopic findings investigated were diffuse atrophy (open type), fundal atrophy, map-like redness, patch redness, erosions (body/antrum), hematin, endoscopic reflux esophagitis, hematin, and duodenitis.

**Results:**

In univariate analysis, diffuse atrophy, map-like redness, and patchy redness at antrum after *H. pylori* eradication were statistically different. In multivariate analysis, map-like redness [*p* < 0.01, 95% confidence interval (CI) 1.83–7.59] and patchy redness at antrum (*p* = 0.02, 95% CI 1.11–4.74) observed were statistically more frequent in patients with synchronous-metachronous gastric dysplasia.

**Conclusion:**

Map-like redness and patchy redness are newly identified endoscopic risk factors for gastric cancer in patients treated for *H. pylori* eradication.

## Introduction

Gastric cancer remains a critical global health burden, particularly in high-incidence regions like Korea, ranking among the leading causes of cancer-related mortality worldwide. The identification of *H. pylori* as the primary risk factor revolutionized understanding of gastric carcinogenesis. *H. pylori* is a Gram-negative, spiral-shaped bacterium transmitted primarily through fecal-oral and oral-oral routes, often during childhood, and uniquely adapted to colonize the hostile acidic environment of the human stomach through its production of urease (1). This chronic infection affects approximately half of the world’s population and is associated with a wide spectrum of gastroduodenal diseases, including peptic ulcer disease, MALT lymphoma, and gastric adenocarcinoma (2). The International Agency for Research on Cancer (IARC) classified *H. pylori* as a definitive Class I carcinogen in 1994 (3), solidifying its role in initiating the multi-step histological progression known as the Correa cascade (4).

*Helicobacter pylori* infection is closely related to the development of gastric cancer. Therefore, it is thought that *H. pylori* eradication treatment may reduce the risk of stomach cancer, but it is not clear how much eradication can reduce the risk. Previous studies have shown that it does not reduce the mortality associated with stomach cancer (5), but this is still unclear. A recently published meta-review has searched for randomized controlled trials (RCTs) and performed a final analysis of a total of 10 RCTs. The analysis focused on seven studies involving 8,323 healthy people and found that eradication treatment significantly reduced the risk of gastric cancer (RR = 0.54; 95% CI 0.40–0.72; NNT = 72) and gastric cancer mortality (RR = 0.61; 95% CI 0.40–0.92; NNT = 135) in healthy people (6). However, when analyzing 7,079 subjects from five studies that presented overall mortality, overall mortality was not reduced (RR = 0.97; 95% CI 0.85–1.12) by *H. pylori* eradication. To summarize the main results, eradication therapy was reported to reduce the risk of gastric cancer by 34% in patients with asymptomatic infection (RR = 0.66; 95% CI 0.46–0.95), but unfortunately, there was no conclusive evidence showing that eradication therapy reduced the incidence of gastric cancer in patients with atrophic gastritis or intestinal metaplasia (5).

We emphasize that gastric cancer screening endoscopy is necessary because eradication therapy does not prevent all gastric cancers. Although it decreases somewhat after eradication, most atrophic gastritis remains, and it has been predicted that genetic abnormalities will occur due to its high regenerative activity and rapid cell turnover, which will lead to dysplasia and subsequent cancer. A recent retrospective cohort study of 2,737 patients who underwent annual endoscopic follow-up after treatment of *H. pylori* infection reported that the longer the follow-up period, the greater the risk of developing diffuse gastric cancer in patients with mild to moderate atrophy at baseline (7). In the study, the incidence of gastric cancer was 0.35% per year after *H. pylori* eradication. Based on these results, there was an opinion that eradication treatment of *H. pylori* would not have a preventive effect on gastric cancer after chronic gastritis progressed (point-of-no return theory) (8). Therefore, the importance of endoscopic follow-up examinations is increasing, and methods to select high-risk groups or set alarm findings and induce careful observation based on specific endoscopic findings appear to be necessary. As eradication treatment has been ongoing for more than 30 years, most gastric cancers are now likely to occur in the post-eradication state. This study aimed to find meaningful results by gathering characteristic mucosal responses after successful *H. pylori* eradication treatment in high-risk patients with gastric cancer who presented with synchronous/metachronous lesions.

## Materials and methods

**Figure 1:**
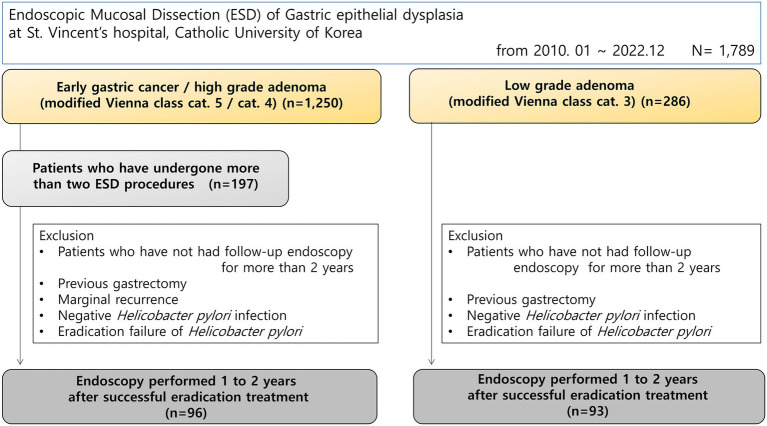
Study flowchart.

This retrospective cohort study was conducted at the Division of Gastroenterology, Department of Internal Medicine, St. Vincent’s Hospital, College of Medicine, The Catholic University of Korea. We reviewed the medical records and endoscopic databases of consecutive patients who underwent endoscopic submucosal dissection (ESD) for gastric epithelial dysplasia between March 2010 and December 2022. During the study period, a total of 1,789 patients underwent ESD for gastric dysplasia at our institution. These patients were categorized these groups based on histological diagnosis: (1) early gastric cancer or high-grade dysplasia (modified Vienna classification categories 4 or 5, *n* = 1,250), and (2) low-grade dysplasia (modified Vienna classification category 3, *n* = 286) (9). An additional 253 patients had other diagnoses or incomplete classification data. From the early gastric cancer/high-grade dysplasia group (*n* = 1,250), we identified 197 patients who underwent two or more ESD procedures for synchronous or metachronous lesions, representing a high-risk population for gastric cancer. Only patients with confirmed *H. pylori* infection at the time of the procedure, who received eradication treatment and were followed endoscopically for at least 2 years were included. Patients with recurrence in the periphery of the previous procedure were excluded. The control group included patients who received endoscopic treatment for simple low-grade dysplasia, were confirmed to have *H. pylori* infection at the time of procedure, and were followed for at least 2 years (Figure 1). Patients who had gastrectomy surgery, negative *H. pylori* infection status, eradication refusal or failure were also excluded. This study was approved by the Institutional Review Board of St. Vincent’s Hospital, Catholic University of Korea (approval number: VC24RISI0243).

### Definition of *Helicobacter pylori* infection and eradication

The diagnosis of *H. pylori* infection was based on two or more positive results from tests including the rapid urease test (RUT), polymerase chain reaction (PCR), urea breath test (UBT), histologic evidence by silver staining, and a serologic test. Two biopsies were used for RUT (CLO® test; Kimberly-Clark, UT, USA). DNAs were extracted from specimens using QIAamp DNA Mini kit (Qiagen, Valencia, CA, USA) according to the manufacturer’s protocol, and stored at −20 °C until use. PCR (Seeplex® ClaR-*H. pylori* ACE Detection; Seegene Institute of Life Science, Seoul, Korea) was performed to identify point mutation-containing gene fragments, according to the manufacturer’s recommendations. A ^13^C-UBT was performed with a Pranactin-Citric drug product, a component of the BreathTek UBT Kit (Korea Otsuka Pharmaceutical Co. Ltd., Seoul, Korea). Three grams of reconstituted Pranactin-Citric containing 75 mg of ^13^C-urea was ingested by the patient. Breath samples before and 20 min after the administration of ^13^C-urea were collected after a mouthwash. The ^13^C/^12^C ratio in the breath samples was measured with an infrared spectrophotometer (UBiT-IR300; Korea Otsuka Pharmaceutical Co. Ltd.). Changes in ^13^C value over baseline were expressed as Δ^13^C, and a positive result was defined as an increase of >2.5%. The presence of serum immunoglobulin G antibodies against *H. pylori* (Enzygnost®, Dade Behring, Marburg, Germany) was determined. An antibody titer >15 U/mL was classified as *H. pylori* seropositive, whereas a titer <10 U/mL was considered as *H. pylori* seronegative accordance with the manufacturer’s instructions. All these tests were performed at the Department of Laboratory Medicine, St. Vincent’s Hospital, as part of routine clinical practice during the study period.

Eradication therapy was performed according to the Korean guideline for *H. pylori* infection (2020 revision) (10), and eradication success was confirmed by UBT at least 4 weeks after completion of therapy. If *H. pylori* test was positive within 1 year after eradication (recrudescence) or after 1 year (reinfection) during follow-up period, either patient was excluded.

### Definition of synchronous and metachronous gastric epithelial dysplasia

In this study, patients were classified as marginal, synchronous, or metachronous lesions. A synchronous lesion was defined as a lesion found within 12 months other than a concurrent gastric epithelial lesion found on ESD. A metachronous lesion was defined as a recurrence more than 12 months after the initial ESD in a lesion that was not connected to the primary lesion and was located more than 1 cm away (11).

### Evaluation of endoscopy

**Figure 2:**
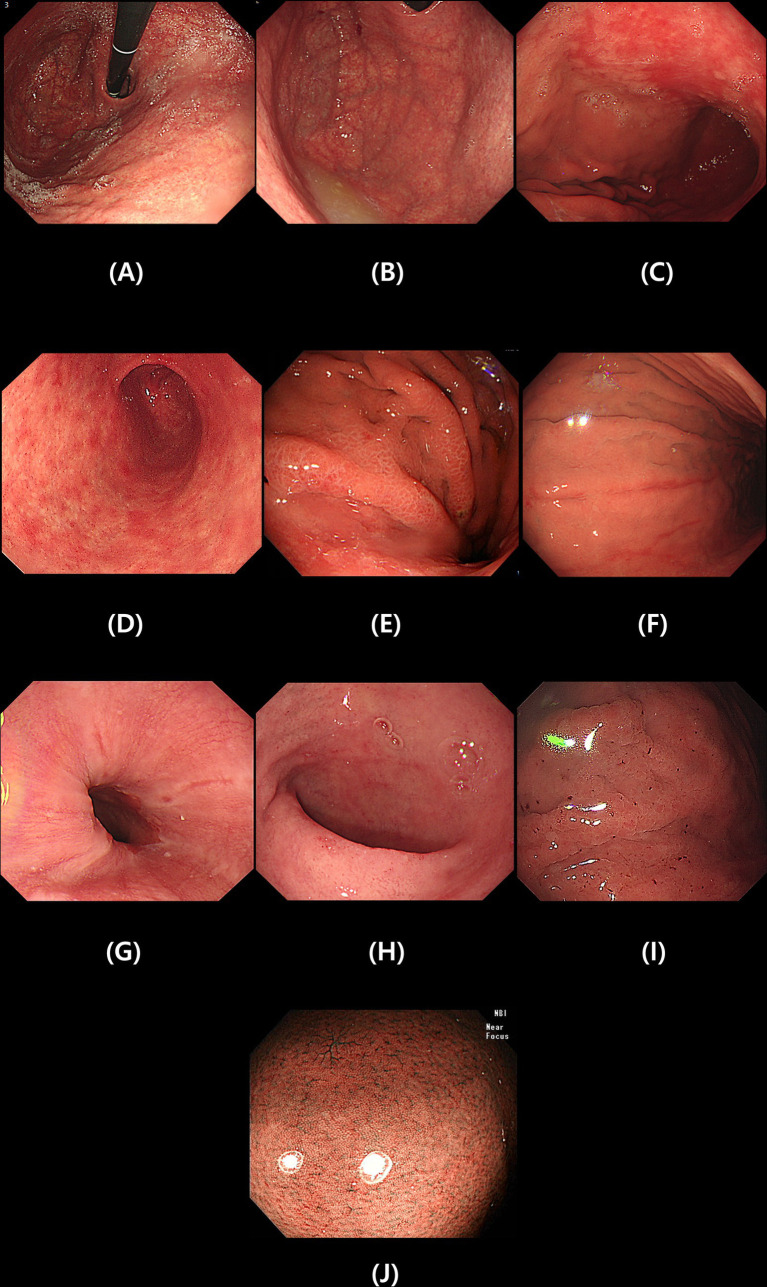
Specific endoscopic findings after *Helicobacter pylori* eradication. Diffuse atrophy **(A)**, Fundal atrophy **(B)**, Map-like redness **(C)**, Patchy redness at antrum **(D)**, Mucosal edema **(E)**, Erosion **(F)**, Reflux esophagitis **(G)**, Duodenitis **(H)**, Hematin **(I)** and Restoration of regular arrangement of collecting venules **(J)**.

Esophagoduodenoscopy was performed using a GIF-XQ 260 (Olympus Medical, Inc. Seoul, Korea). We investigated whether endoscopy was performed between 1 and 2 years after successful eradication of *H. pylori*. Endoscopic examinations reviewed all images in picture archiving and communication system (PACS) by two different endoscopists (Chung WC; yearly >1,000 diagnostic endoscopies, 20 years, Lim NR; yearly 1,000 diagnostic endoscopies, 5 years). Disagreement between the two examiners made an exclusion, and we evaluated specific endoscopic findings – diffuse atrophy (open type), fundal atrophy, map-like redness, patchy redness at antrum, erosions (body/ antrum), mucosal edema, hematin, reflux esophagitis, and duodenitis (12, 13). We also checked the restoration of regular arrangement of collecting venule (RAC) pattern (14). Figure 2 showed clinical photos of endoscopic findings.

1. Diffuse atrophy (open type)—the classification of diffuse atrophic gastritis was defined as open type if it included gastric cardia according to the Kimura-Takemoto classification (Figure 2A) (15).
2. Fundal atrophy—in cases where atrophy of the gastric glands progresses from the body side to extensive fundal atrophy is observed (Figure 2B).
3. Map-like redness—in gastric atrophy, the improvement pattern is different between the area with only inflammation and the area with atrophy after *H. pylori* eradication treatment. In this case, map-like redness is observed in the gastric body (Figure 2C) (16, 17).
4. Patchy redness at antrum—the patchy redness seen after *H. pylori* eradication often has a clear border and a slightly sunken form, and sometimes it is accompanied by extensive map-like erythema in the gastric body. It is also associated with intestinal metaplasia (Figure 2D) (16).
5. Mucosal edema—soft, thick, and distended mucosa (Figure 2E).
6. Erosion (body/antrum) (Figure 2F).
7. Reflux esophagitis—patchy, striated, or circular epithelial defects in the mucosa in the distal esophagus. We also included erythema, blurred junction between squamous epithelium and columnar epithelium, and increased vascular marking in the distal esophagus as minor changes (Figure 2G).
8. Duodenitis—erythematous or exudative change with erosions in the bulb or post bulbar area (Figure 2H).
9. Hematin—red or dark ecchymosis spots or flecks (Figure 2I).
10. Restoration of regular arrangement of collecting venules (RAC). When the gastric mucosa of the gastric body without atrophy is observed using a magnifying endoscope and narrow band image, collecting venules appear as numerous, uniformly sized, fine red dots at regular intervals in the gastric body. This finding is called a RAC (Figure 2J) (14).

### Statistics

All statistical analyses were performed with the SPSS program (SPSS version 25.0; IBM, Chicago, IL, USA). Values are expressed as mean ± standard deviation. Categorical variables were presented as numbers with percentages and were tested by a Chi-square test or Fisher’s exact test. Multivariate logistic regression was performed to identify independent risk factors. *p*-value <0.05 was considered significant.

## Results

Between March 2010 and December 2022, 1,789 consecutive patients underwent endoscopic submucosal dissection (ESD) for gastric epithelial dysplasia at our institution. Based on histological diagnosis using the modified Vienna classification, patients were categorized as follows: 1,250 patients had early gastric cancer or high-grade dysplasia and 286 patients had low-grade dysplasia. Among patients with early gastric cancer of high-grade dysplasia, we identified patients who had synchronous or metachronous gastric dysplasia at high risk for gastric cancer and underwent mucosal dissection more than twice. 96 patients met all criteria and were enrolled in the study. Among 286 patients who underwent gastric mucosal resection or submucosal dissection for simple low-grade gastric epithelial dysplasia, the control group consisted of 93 patients diagnosed with *H. pylori* infection at the time of the procedure, successfully treated for eradication, and followed up for more than 2 years. However, patients who refused eradication treatment or did not achieve successful eradication even after third-line eradication treatment were excluded from the analysis.

There were no significant differences in the patients’ baseline age, gender, smoking history, or drinking history. In univariate analysis, diffuse atrophy, map-like redness, and patchy redness at antrum after *H. pylori* eradication were significantly different (Table 1). The frequency of recovery of RAC pattern was 80.4% in synchronous-metachronous gastric dysplasia group and 85.2% in control group, with no difference between the two groups. There was no statistically significant difference in the presence or absence of diffuse atrophy or fundal atrophy in synchronous-metachronous gastric dysplasia group compared to the control group, which were previously known as risk factors. In multivariate analysis, map-like redness [*p* < 0.01, 95% confidence interval (CI) 1.83–7.59] and patchy redness at antrum (*p* = 0.02, 95% CI 1.11–4.74) observed were found to be significantly more frequent in patients with synchronous-metachronous gastric dysplasia compared to control group (Table 2). Map like redness and patchy redness at antrum were often observed simultaneously. When these two items were combined into one multivariate analysis, the statistical difference was more evident (*p* < 0.01, 95% CI 2.248–8.918). When only the 45 patients classified as patients with isogenic dysplasia in this study were analyzed separately, map-like erythema (*p* < 0.01, 95% CI 1.69–10.89) and irregular erythema of the stomach (*p* = 0.01, 95% CI 1.34–8.53) were observed frequently in target patients. (Table 3).

**Table 1:** Baseline demographics of the patients.

Variables	Synchronous / Metachronous Gastric epithelial dysplasia (*N* = 96)	Low grade dysplasia (*N* = 93)	*p* value
Age (years)	64.86 ± 11.97	65.95 ± 12.54	0.42
Male sex (%)	65 (67.7)	68 (73.1)	0.42
Female sex (%)	31 (32.3)	25 (26.9)	
Current smoker	39 (40.6)	38 (40.9)	0.97
Alcohol drinking	53 (55.2)	57 (61.3)	0.40
Early gastric cancer	39		
High grade dysplasia	57		
Metachronous recurrence	47		
Diffuse atrophy (open type)	61 (63.5)	43 (46.2)	<0.01*
Fundal atrophy	27 (28.1)	22 (23.7)	0.48
Map like redness	51 (53.1)	21 (22.6)	<0.01*
Patchy redness at antrum	39 (40.6)	21 (22.6)	<0.01*
Erosions (antrum)	20 (20.8)	20 (21.5)	0.91
Erosions (body)	26 (27.1)	19 (20.4)	0.29
Mucosal edema	19 (19.8)	12 (12.9)	0.20
Hematin	26 (27.1)	30 (32.3)	0.44
Reflux esophagitis	31 (32.3)	21 (22.6)	0.14
Duodenitis	7 (7.3)	8 (8.6)	0.74
Restoration of regular arrangement of collecting venules (RAC)	45/ 56 (80.4)	23/ 27 (85.2)	0.59

Values are presented as mean ± standard deviation or number (%).

Closed, Open; classified by Kimura Takemoto endoscopic classification.

Statistical comparisons were performed using Student *t*-test for continuous variables and Chi-square test or Fisher’s exact test for categorical variables.

*Statistically significant.

**Table 2:** Results of a multivariate analysis comparing mucosal changes after *Helicobacter pylori* eradication in patients with simultaneous / metachronous gastric epithelial dysplasia with those in patients with low-grade dysplasia.

	Odds ratio (OR)	95% confidence interval (CI) Low Upper	*p* value
Age (years)	0.995	0.954 ~ 1.038	0.813
Male sex (%)	0.612	0.267 ~ 1.307	0.204
Current smoker	0.595	0.245 ~ 1.443	0.251
Alcohol drinking	0.965	0.404 ~ 2.307	0.936
Diffuse atrophy (open type)	1.102	0.469 ~ 2.586	0.824
Fundal atrophy	1.227	0.553 ~ 2.722	0.615
Map like redness	3.724	1.827 ~ 7.591	<0.001*
Patchy redness at antrum	2.297	1.113 ~ 4.744	0.025*
Erosions (antrum)	1.172	0.499 ~ 2.750	0.716
Erosions (body)	1.570	0.728 ~ 3.342	0.242
Mucosal edema	1.253	0.508 ~ 3.090	0.624
Hematin	0.748	0.360 ~ 1.557	0.438
Reflux esophagitis	1.318	0.630 ~ 2.775	0.463
Duodenitis	0.636	0.183 ~ 2.203	0.475

Variables in univariate analysis were included in the multivariate logistic regression model.

OR, odds ratio; CI, confidence interval.

*Statistically significant.

**Table 3:** Results of a multivariate analysis comparing mucosal changes after *Helicobacter pylori* eradication in patients with metachronous gastric epithelial dysplasia (*n* = 45) with those in patients with low-grade dysplasia.

	Odds ratio (OR)	95% confidence interval (CI) Low Upper	*p* value
Age (years)	1.026	0.969 ~ 1.087	0.376
Male sex (%)	0.608	0.216 ~ 1.708	0.345
Current smoker	0.695	0.213 ~ 2.270	0.547
Alcohol drinking	0.618	0.179 ~ 2.134	0.447
Diffuse atrophy (open type)	1.573	0.477 ~ 5.180	0.457
Fundal atrophy	1.051	0.343 ~ 3.215	0.931
Map like redness	4.295	1.694 ~ 10.887	0.002*
Patchy redness at antrum	3.386	1.336 ~ 8.583	0.010*
Erosions (antrum)	1.045	0.322 ~ 3.389	0.941
Erosions (body)	0.499	0.802 ~ 6.366	0.123
Mucosal edema	0.859	0.127 ~ 1.960	0.319
Hematin	0.959	0.370 ~ 2.485	0.931
Reflux esophagitis	1.171	0.436 ~ 3.146	0.754
Duodenitis	1.274	0.294 ~ 5.508	0.746

Variables in univariate analysis were included in the multivariate logistic regression model.

OR, odds ratio; CI, confidence interval.

*Statistically significant.

## Discussion

This study identified map like redness and patchy redness at antrum observed after *H. pylori* eradication as novel endoscopic features associated with synchronous or metachronous gastric dysplasia in high-risk patients. These findings were statistically significant after adjusting for known risk factors including the extent of atrophic gastritis. The odds ratios were substantial, with a 3.7-fold increased risk for map like redness and a 2.3-fold increased risk for patchy redness at antrum. Importantly, when either finding was present, the odds ratio increased to 4.5, suggesting these markers can effectively identify a high-risk population requiring intensive surveillance.

This persistent cancer risk aligns with existing clinical evidence. When eradication therapy is performed, it has been reported that the effect of eradication therapy in suppressing the occurrence of cancer can be maximized in the absence of precancerous lesions (atrophic gastritis, intestinal metaplasia, or dysplasia) (8). Atrophic gastritis is a chronic inflammatory change in the gastric mucosa, which occurs in association with *H. pylori* infection, various environmental factors, and autoimmune responses to gastric glandular tissue. The decrease in the gastric glandular tissues in the gastric body due to this atrophic change causes hypoacidity, and the resulting change in the gastric environment causes a decrease in ascorbic acid in the stomach and bacterial overgrowth, which induces the production of N-nitroso compounds, an important mechanism for the occurrence of gastric cancer (4). However, the reversibility of advanced precancerous lesions remains inconsistent. While some studies observed an improvement in atrophic changes and intestinal metaplasia after *H. pylori* eradication, others reported no changes or even significant progression of intestinal metaplasia, suggesting limited efficacy (18, 19). A clinical study conducted in Japan followed up patients with atrophic gastritis infected with *H. pylori* for 5 years after eradication treatment (20). The results showed that atrophic changes and intestinal metaplasia improved in both the gastric body and the gastric antrum, but this was highly correlated with high blood levels of pepsinogen I. Since there is a lack of specific tests or findings that can determine the degree of atrophic gastritis or intestinal metaplasia before eradication, there is a possibility that atrophic gastritis or intestinal metaplasia that has already progressed significantly may continue to progress along with genetic changes even after eradication.

The molecular mechanisms underlying this persistent cancer risk can be understood through the stepwise transformation process of the Correa cascade (4). *H. pylori* infection leads to a dysregulated immune response characterized by the infiltration of inflammatory cells in the tumor microenvironment, thus secrete numerous inflammatory cytokines, chemokines, and reactive oxygen species/reactive nitrogen species, resulting in apoptosis, genomic instability, and the development of gastric cancer (21–23). These chronic inflammatory processes induce widespread epigenetic modifications, most notably DNA hypermethylation of tumor suppressor genes. As inflammation progresses to atrophic gastritis, glandular loss leads to hypochlorhydria and bacterial overgrowth, which facilitates the production of N-nitroso compounds. These molecular damage is imprinted in the genome of premalignant lesion (intestinal metaplasia and dysplasia), which represents a “point of no return” (24). Therefore, while eradication eliminates the bacterial initiator, the accumulated non-reversible damage in the mucosa—reflected by persistent endoscopic findings such as map-like redness and patchy redness—remains the core driver of long-term risk for gastric adenocarcinoma.

The Operative Link on Gastritis Assessment (OLGA) system was designed to identify the degree of atrophic gastritis and intestinal metaplasia that persists after *H. pylori* eradication (25, 26). This system calculates the risk of gastric cancer by adding endoscopic findings to the histological semiquantitative evaluation of the existing Sydney system and classifies the risk of gastric cancer by considering the location and degree of inflammation in the antrum and body. Low OLGA stages (0, I, and II) reflect low gastric cancer risk, and high stages (III and IV) are high gastric cancer risks. The existing OLGA system included too many high-risk groups in areas with high *H. pylori* prevalence, and a more selective system was required. High interobserver agreement was reported for the Operative Link on Gastric Intestinal Metaplasia Assessment (OLGIM), which utilizes the degree of intestinal metaplasia (27). However, these histology-based systems face practical challenges in routine clinical practice. They require a minimum of 5 tissue biopsies and a maximum of 12 specimens must be collected basically, posing a significant burden in practice. In regions with high *H. pylori* prevalence, a large proportion of patients fall into high-risk categories, limiting the systems’ discriminatory value (28). Moreover, the relatively small number of prospective studies reporting that usefulness of systems is limited (29, 30). Clinicians have called for the development of methods to grade and assess the risk of gastric cancer based solely on endoscopic findings without requiring a biopsy.

In this respect, the Kyoto classification system of gastritis is very attractive to clinicians because it allows them to make judgments based only on endoscopic findings without tissue examination (12). However, the usefulness of the risk of gastric cancer according to the Kyoto classification of gastritis needs to be continuously verified, and the score or items may need to be changed depending on the results. In addition, this classification system is a system that judges at the time of performing an endoscopy and hopes for an immediate solution, and a considerable period of training may be required to understand this and apply this system during an endoscopy (31, 32), so there may be controversy over its usefulness. To alleviate these concerns, it is necessary to identify high-risk groups in the field of endoscopy by presenting more sensitive, strong, and rational opinions, and then induce close observation and tissue examination.

After *H. pylori* eradication treatment, endoscopic findings of the gastric mucosa generally improve, which is a change that occurs as chronic inflammation decreases, and histologically, polynuclear cell infiltration and mononuclear cell infiltration improve. This improvement in the inflammatory response appears over a long period of time, from immediately after eradication treatment to several years (33). After successful eradication, punctate erythema, diffuse erythema, mucosal edema, hypertrophy of the gastric folds, and turbid mucus often improve (34). It is also known that the uniform vascular pattern reappears in the background mucosa and the border of atrophy becomes unclear. Among the findings observed after eradication, map like redness is a highly specific lesion. Histologically, it corresponds to intestinal metaplasia. The mechanism by which map like redness appears can be explained by the disappearance of diffuse erythema after successful eradication treatment and the enhancement of the contrast between non-atrophic mucosa and atrophic mucosa (16).

This study is to investigate whether there were characteristic mucosal changes after eradication in high-risk groups related to the occurrence of gastric cancer. Therefore, we focused on the unique mucosal changes that appear after eradication in patients with simultaneous or metachronous early gastric cancer or precancerous lesions, and among them, map like redness and patchy redness at antrum were significant findings. It is noteworthy that the odds ratio was approximately 4.48, when even one of these two lesions appeared. When limited to metachronous lesions, the values were 4.30 and 3.39, respectively, which suggests that cases with these findings can be considered a high-risk group.

Several limitations warrant acknowledgment. First, this was a single-center retrospective study, which may limit generalizability to other populations and healthcare settings. Our findings require validation in independent, preferably multi-center prospective cohorts. Second, we did not systematically perform biopsies according to standardized protocols to correlate endoscopic findings with histological severity of atrophy and intestinal metaplasia. Such correlation would strengthen understanding of the biological basis of map-like redness and patchy redness and their relationship to underlying mucosal pathology. Third, inter-observer variability for recognizing map-like redness and patchy redness was not formally assessed, although we addressed this by excluding cases with disagreement between reviewers. Fourth, the relatively short follow-up period may not capture all cases of metachronous gastric cancer, as some cancers may develop after longer intervals. Fifth, both study and control groups consisted of patients with dysplasia who had undergone ESD, representing a selected risk population. This design allowed us to compare very high-risk patients (synchronous/metachronous lesions) with relatively low to moderate risk patients (single low-grade dysplasia), but limits assessment of how these markers perform in screening populations. Finally, we did not assess the severity, extent, or specific anatomical distribution patterns of map-like redness and patchy redness, which might provide additional prognostic information.

In conclusion, this study, based on 12 years of data from a substantial patient cohort, demonstrates that map-like redness and patchy redness at antrum observed after *H. pylori* eradication are independent endoscopic risk factors for synchronous or metachronous gastric dysplasia in high-risk patients. These findings can be readily recognized during routine endoscopy without requiring biopsies, making them practical tools for risk stratification in clinical practice. Patients exhibiting these endoscopic features may benefit from intensified surveillance strategies including shorter examination intervals and more meticulous evaluation. Our findings address an important clinical need: identifying which patients remain at elevated gastric cancer risk despite successful *H. pylori* eradication. As eradication therapy becomes increasingly widespread and most future gastric cancers will occur in post-eradication patients, practical methods for risk stratification become critical. Map-like redness and patchy redness represent easily recognizable markers that can guide personalized surveillance strategies. However, these findings require validation in independent, prospective, multi-center cohorts before definitive clinical recommendations can be established. With such validation, these endoscopic findings could be incorporated into clinical guidelines to optimize post-eradication surveillance and improve early detection of gastric cancer in high-risk populations.

## Data Availability

The datasets generated or analyzed during the study are available from the corresponding author on reasonable request.
